# A Case Report on Life-Threatening Lower Gastrointestinal Bleeding: A Rare Presentation of Mucinous Adenocarcinoma of the Appendix

**DOI:** 10.1155/2021/2349737

**Published:** 2021-09-21

**Authors:** H. W. T. D. Wijayaratne, K. J. A. Fernando, T. Matheeshan

**Affiliations:** ^1^Postgraduate Institute of Medicine, University of Colombo, Sri Lanka; ^2^Ministry of Health, Sri Lanka

## Abstract

**Introduction:**

A life-threatening lower gastrointestinal bleeding from mucinous adenocarcinoma of the appendix is a rare occurrence. Diagnosing and management of such a condition are challenging. *Case Presentation*. A 73-year-old male with a history of type 2 diabetes mellitus and hypertension presented with intermittent per rectal bleeding for two weeks, which progressed to the passage of a large number of blood clots and fresh blood. He had features of class III shock on admission. An endoscopic evaluation followed initial resuscitation to locate the source of bleeding. Colonoscopy revealed a large blood clot at the opening of the appendicular orifice with no active bleeding. Oesophagoduodenoscopy, contrast-enhanced CT abdomen, and CT angiogram findings were unremarkable. Due to repeated episodes of rebleeding leading to haemodynamic instability, an exploratory laparotomy was performed. A retrocaecal appendix was noticed with a macroscopically suspicious-looking dilated tip adhered to the posterior caecal wall. Right hemicolectomy was performed as the lesion was suspicious and to stop bleeding from the site. Ileocolic side-to-side hand-sewn anastomosis was performed using 3/0 polyglactin. Postoperatively, per rectal bleeding was settled. Microscopy revealed appendiceal mucinous adenocarcinoma with AJCC staging of pT3NoMx. The patient was discharged on postoperative day seven and referred to oncological management. He was offered six cycles of chemotherapy with capecitabine and oxaliplatin. At the six-month follow-up visit, the patient had no features of recurrence clinically.

**Conclusion:**

Mucinous adenocarcinoma of the appendix can rarely present as life-threatening lower GI bleeding. Prompt resuscitation, endoscopic evaluation, and operative management with right hemicolectomy and chemotherapy provided a good outcome.

## 1. Introduction

Gastrointestinal (GI) bleeding is associated with significant mortality and morbidity. It has an in-hospital all-cause mortality of 3.9% and accounted for the second most common cause for blood transfusion in hospitals in the UK [1]. Lower GI bleeding accounts for ¼ of the total number of cases of all GI bleeding.

Causes for lower GI bleeding could be either due to benign causes or malignant causes. Common benign causes are diverticular disease, angiodysplasia, and colitis [2]. Malignant causes include colonic malignant neoplasms. A nationwide audit carried out in the UK showed that 6.1% of all lower gastrointestinal bleeding are due to malignancies in the colon to the anal canal. The severity of bleeding varies from mild to life-threatening severe bleeding. The above study showed that presentation of acute life-threatening lower gastrointestinal bleeding with shock was uncommon and was around 2.3% of all cases [3].

It is a rare occurrence that a patient presents with an acute life-threatening lower gastrointestinal bleeding as a result of severe bleeding from an appendiceal mucinous neoplasm of which we discuss in this case report.

## 2. Patient Information

A 73-year-old male with a history of type 2 diabetes mellitus and hypertension presented with intermittent per rectal bleeding for two weeks duration and passage of a large number of clots and fresh blood in one day.

On admission, he was pale and haemodynamically unstable and was in class III shock. The patient was managed as an emergency and started resuscitation. He was given high-flow O_2_ via a face mask. A wide bore IV cannula was inserted, and initial fluid resuscitation was done with 0.9% saline 500 ml and one pack of uncrossed matched O negative blood transfusion. Later, he was transfused with cross-matched “A” positive red cell concentrates (RCC) until the patient's haemodynamic parameters were stable. Altogether, five packs of RCC were given on the first day. He was monitored closely for the 1^st^ 24 hours.

Initial haematological investigations revealed haemoglobin of 4.9 g/dl. His platelet count was 399 × 10^3^ with PT/INR of 11.9/1.04. The liver enzyme study was within the normal range.

The patient had intermittent per rectal bleeding and anaemia for the past two years and was evaluated on two occasions. Lower gastrointestinal endoscopy was insignificant, and faecal occult blood was negative. He had been managed only with haematinics. The persistence of anaemic symptoms led him to seek medical advice again. Oesophagogastroscopy (OGD) revealed several duodenal ulcers and was treated with *H. pylori* eradication treatment. The follow-up endoscopy showed healed duodenal ulcers and an absence of active disease.

The patient was not on antiplatelet or anticoagulants. Regular medications were enalapril 5 mg bd for hypertension, metformin 1 g bd, and glibenclamide 5 mg tds for diabetes mellitus. He had a fair glycaemic control.

On physical examination, he did not have stigmata of chronic liver cell disease. Abdominal examination revealed a mildly distended abdomen with no organomegaly or intra-abdominal masses. Fresh per rectal bleeding with blood clots was noticed during the digital rectal examination, and there was no palpable rectal wall growth. Systemic examination was normal.

An urgent OGD was carried out suspecting a massive upper GI bleeding as he had a history of duodenal ulcers two years back. However, it only revealed features of mild gastritis and any active bleeding point was not identified from the oesophagus to the 2^nd^ part of the duodenum. A colonoscopy was planned. On the first day of admission, he did not develop a rebleed, and he was haemodynamically stable with initial resuscitation.

**Figure 1:**
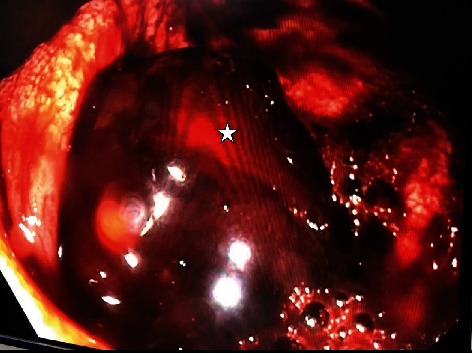
Image from lower GI endoscopy. The star indicated the blood clot at the appendicular opening in the caecum.

On the 2^nd^ day of admission, a colonoscopy was performed. The patient was fasting since the admission, and preparation for the colonoscopy was done only using an enema. The examination was carried out up to the terminal ileum. The anal canal was normal. The wall of the whole rectum and the colon was covered with altered blood. Any active bleeding point was not noticed until the caecum. The wall of the rectum and colon was visualised properly by injecting water through the biopsy port. There was a mucous plug mixed with altered blood adhered to the appendicular opening Figure 1. The clot was not dislodged with water injection, and active bleeding was not noticed. The ileum was devoid of blood. So the suspicion was pointed towards the appendix and caecal region.

As both upper and lower GI endoscopy findings were inconclusive, a CT angiogram along with a contrast-enhanced computed tomography (CECT) abdomen was performed to locate a specific bleeding point. Any contrast material leak was not noticed in the stomach, small bowel, ileocaecal region, or large bowel up to the rectum and was not identified in CT angiogram. It is assumed that the false-negative result could be due to a sealed bleeding point when performing the investigation. Contrast-enhanced CT abdomen did not reveal any anatomical abnormality in the GI tract. The ileocaecal region was explicitly examined for abnormalities as the colonoscopy finding was suspicious in the ileocaecal region. Except for a retrocaecal appendix, any mass or vascular malformation was not identified by the radiology team, which consisted of an interventional radiologist. The liver was examined for secondaries, and the liver appeared healthy.

On the third day of the admission, the patient developed intermittent episodes of per rectal bleeding with hypotensive episodes. He was managed with transfusion of blood and blood products. Meanwhile, a thromboelastogram (TEG) was performed to detect any coagulopathy, and it showed low platelet activity. Five units of platelets were transfused following the results of the thromboelastogram. Then, the decision has been taken to explore the abdomen to find the cause and arrest the bleeding. An emergency exploratory laparotomy was performed on the night of the third day after optimisation of haemodynamic and haematological parameters.

**Figure 2:**
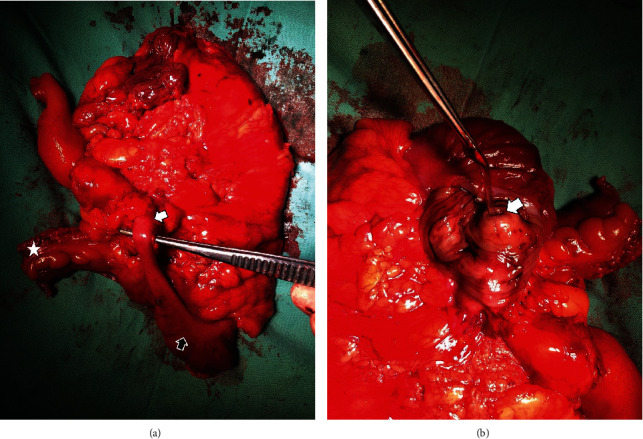
(a) Macroscopic appearance of the posterior aspect of the right hemicolectomy specimen. The white arrow shows the tip of the appendix. The black arrow shows the base of the appendix and the caecum. The white star represents the terminal ileum. (b) Macroscopic appearance of the split and opened caecum. The white arrow shows the mucinous material inside the lumen of the appendix.

A lower midline laparotomy incision was made. A retrocaecal appendix was noted with its dilated tip adhered to the caecal wall. The serosa of the appendix was not ruptured. There was neither a growth nor inflammation in the caecum and surrounding tissue. Any noticeable mesenteric lymph node mass was not detected. The parietal peritoneum was smooth, and the peritoneal cavity was normal. Peritoneal deposits were not noticed. Free fluid or palpable hepatic metastasis was not observed. The finding of the rest of the gastrointestinal tract was insignificant. Considering the collective evidence from the colonoscopy and the surgery findings, a malignant lesion in the appendix was suspected, and intraoperative decision was made to perform a right hemicolectomy. An ileocolic side-to-side hand-sewn anastomosis was performed using 3/0 polyglactin. Care was taken not to spill mucin to the peritoneal cavity. The peritoneal cavity was washed well with distilled water, and a 28 F IC tube drain was applied. When examining the specimen, it was noticed that the lumen of the appendix was comprised of mucinous material mixed with blood (Figures 2(a) and 2(b)). The specimen was sent for histological analysis. Postoperatively, the patient was stable.

On postoperative day 2, the patient's per rectal bleeding was settled and passed normal colour stools. A superficial surgical site infection was managed with drainage of the superficial pus collection and IV co-amoxiclav 1.2 g 8 hourly and IV metronidazole 500 mg 8 hourly for three days.

The histological diagnosis was appendiceal mucinous adenocarcinoma. Sections of the appendix revealed an invasive tumour comprised of complex glandular structure and pools of mucin with floating cells. These glands were lined by dysplastic tall columnar epithelial cells. The mucinous area comprises >50% of the tumour. There was no lymphovascular invasion or perineural invasion. Tumour had infiltrative margins. It has invaded through muscularis propria into subserosa and <1 mm away from the serosa. Appendicular attachment to the caecum showed the tumour. Eight lymph nodes removed from periappendicular fatty tissue had only reactive changes. The proximal and distal resection margin was free of the tumour. Pathological staging was pT3NoMx.

The patient was discharged on postoperative day seven. On discharge, he did not have rectal bleeding or melaena. After a total of 10 units of RCC transfusion, haemoglobin was 10.0 g/dl. He was referred for oncological management and was offered six cycles of chemotherapy with capecitabine and oxaliplatin.

Postoperatively, he was assessed for six months and had not developed any episodes of per rectal bleeding. He is currently able to carry out his activities of daily living and functions normally.

## 3. Discussion

Lower gastrointestinal bleeding is defined as bleeding into the gastrointestinal lumen below the level of ligament of Treitz. Out of all gastrointestinal bleeding, 20%–24% of acute gastrointestinal bleeding is due to lower GI bleeding, which is three times lesser than upper GI bleeding [4, 5].

Lower gastrointestinal bleeding patients may present with sudden fresh bleeding per rectum (haematochezia) or as melaena [5]. Melaena occurs when it is originating in the right colon or proximally. Massive bleeding originating from an upper GI source similarly can present as haematochezia [6].

When a patient is presented to the emergency department with life-threatening per rectal bleeding, the main steps of management include quick assessment and hand-in-hand resuscitation of the patient. Correction of any coagulopathy, finding the anatomical and pathological cause for the bleeding, and treating the cause are also mandatory. Then, the patient should be monitored for early detection of complications.

The British Society of Gastroenterology has developed guidelines on diagnosis and management of acute gastrointestinal bleeding [7]. Initial management of an unstable patient presenting with acute lower GI bleeding includes assessment and concurrent airway management, breathing, and circulation. Resuscitation with crystalloid and blood products with correction of any coagulopathy is paramount. Most lower GI bleeds settle with the above measures. In this case, the patient was haemodynamically stable after initial resuscitation on the first day. However, he began to rebleed on the following days. In a study done by Oakland and colleagues, about 11.0% of lower GI bleeding cases continue to bleed within 24 hours of admission, and about 13.6% of patients had one or more rebleeding episodes following the admission [3]. This implicates that even though the patient is haemodynamically stabilised following initial resuscitation, frequent monitoring is necessary within the first 24-48 hours for the early detection of a rebleeding.

In the same study, it was found that 29.4% of the study population with lower GI bleeding was on antiplatelets, 23.1% was on aspirin and 10.7% was on warfarin [3]. Inquiry on antiplatelets and anticoagulants in history is essential as therapeutic interventions are needed to reverse the effects of drugs.

The diagnosis of the anatomical and pathological cause is challenging. Imaging studies and endoscopic studies are crucial. CT angiogram and endoscopy are the first-line investigations to diagnose the site of bleeding and underlying pathology, respectively.

Our patient recovered with initial resuscitation on the first day. Considering the availability of multidetector CT angiogram in late hours in our institute and the patient's clinical condition, we decided to do an endoscopic evaluation before a CT angiogram. According to the UK guideline on acute lower GI bleeding, patients with a major lower GI bleeding need to be offered an urgent colonoscopy [7]. Colonoscopy in a patient with suspected lower GI bleeding has a diagnostic and therapeutic value. In cases of lower GI bleeding, the possibility of arriving at a diagnosis varies 42%-90% with colonoscopy. In our case, the abnormality was a blood clot mixed with mucinous material on the appendiceal opening, which directed the suspicion towards the appendicular pathology.

In haemodynamically stable patients with severe lower GI bleeding, urgent oesophagogastroscopy is recommended if clinically indicated. The reason is that 11%-15% of patients who have clinical features of lower GI bleeding are found to have a bleeding point in the upper GI region [7]. During the initial workup, this patient's history of multiple duodenal ulcers pointed towards a possibility of recurrent upper GI bleeding. Thus, an urgent oesophagogastroscopy was carried out to exclude upper GI bleeding. However, the finding was not significant, which led the diagnosis towards a lower GI source.

Multidetector CT angiogram is recommended to diagnose the active bleeding point in haemodynamically unstable patients [7]. Imaging finding of multidetector CT angiogram is active extravasation of contrast medium into the bowel lumen. “Contrast blush” within the lumen of the bowel in the arterial phase is diagnostic. Not only during the arterial phase but the extravasated contrast may also appear in the portal venous phase in cases when the bleeding is slow. Also, there are a variety of contrast appearances depending on their physiological property [8]. Meta-analysis on the accuracy of CT angiography on acute GI bleeding shows about 85.2% (95% CI—75.5% to 91.5%) of overall sensitivity and 92.1% (95% CI—76.7% to 97.7%) of overall specificity [9]. A major pitfall of CT angiogram is false negativity when the patient does not have active bleeding at the time of the test [8]. In our case, following the initial resuscitation, the patient was haemodynamically stable. We specifically looked for any contrast blush around the ileocaecal region and appendix with the help of 2 consultant radiologists. However, the findings were inconclusive at that point. This means the bleeding was intermittent, and the reason for the negative CT angiogram of our patient would be the absence of active bleeding during the CT angiogram study.

In a case of difficulty in diagnosing bleeding point using CT angiogram and endoscopy, nuclear medicine studies such as red cell scintigraphy have a role [7]. It is beneficial when the bleeding rate is slow or intermittent. It uses technetium-99 tagged red blood cells as a radiotracer. Following the radiotracer injection, bleeding can be detected when radiotracer activity is detected outside the usual areas of the blood pool. The advantage of red cell scintigraphy includes high sensitivity in detecting slow bleeding (0.05-0.1 ml/min), assessment of bleeding over a prolonged period, and detection of arterial and venous bleeding [8]. Nonetheless, it has disadvantages like taking a prolonged duration to perform the study and unavailability in all institutes.

There is a place for catheter angiography when the source of bleeding is found using another imaging modality, and the endoscopic measures are failed [8]. Catheter angiography is mainly therapeutic and can perform arterial embolisation to stop bleeding from the identified vessel. However, commonly reported complications are bowel ischemia and the risk of rebleeding. As the diagnosis was uncertain and weighing the risk of bowel ischemia, we did not decide to perform catheter angiogram and embolisation in this patient.

The surgery to control bleeding is indicated only when radiological and endoscopic measures are failed. As our patient developed recurrent bleeds and haemodynamic instability while staying in the ward, we decided to explore the abdomen. Intraoperatively, we could demonstrate a macroscopic abnormality in the appendix. With the collective evidence from endoscopic findings and intraoperative findings, the anatomical site of bleeding was pointed towards the appendix.

Appendiceal neoplasms are rare, and incidence ranges from 0.7 to 1.7%. Nevertheless, recent studies show that there is a rise in the incidence of appendiceal malignancies [10, 11]. These tumours commonly present with features of acute appendicitis or found as an incidental finding during surgery [12].

There are several classification systems to classify appendiceal neoplasms. In the Sri Lankan setup, the pathologists adhere to the WHO classification of tumours of the appendix. In the 5th edition of WHO classification of digestive system tumours, the epithelial neoplasms of the appendix are broadly classified into serrated lesions or polyps, mucinous neoplasms, adenocarcinomas, and neuroendocrine tumours. Mucinous neoplasms are categorised into low-grade mucinous neoplasm or high-grade mucinous neoplasm. Mucinous adenocarcinoma has an infiltrative character and falls into a type of adenocarcinoma [13].

Mucinous adenocarcinoma is the predominant subtype of appendiceal malignant neoplasms found in population-based studies. According to these studies, the mean age of presentation of mucinous adenocarcinoma was 49 years. The prevalence is slightly higher among females compared to males [10, 14]. In the Sri Lankan context, 0.3% of appendicectomy specimen had unexpected mucinous neoplasms [15].

Mucinous lesions of the appendix range from benign to malignant. The significance of mucinous neoplasm is that it can give rise to “pseudomyxoma peritonei (PMP) which is a clinical syndrome in which a mucinous neoplasm grows within the peritoneal cavity causing mucinous ascites and peritoneal implants” [16]. PMP is a prognostically poor condition affecting the peritoneal cavity. Early detection and decision on surgical excision are mandatory to prevent PMP.

Commonly, the mucinous neoplasms of the appendix present as acute appendicitis. When the neoplasm is advanced and complicated with PMP, apart from acute appendicitis, patients can present with features like abdominal distension or new-onset hernia [17]. Bleeding from a mucinous appendiceal neoplasm is rare, and one case report was found in the literature on appendiceal adenocarcinoma presenting with rectal bleeding and haematuria. The tumour mainly involved the distal part of the appendix, small bowel, large bowel, and bladder. Microscopy was partly mucinous adenocarcinoma [18].

Preoperative diagnosis of mucinous neoplasms is difficult on most occasions as mucinous appendiceal carcinoma does not have pathognomonic features. Most of the early detected tumours were incidental findings in the contrast-enhanced CT abdomen performed for RIF pain and some other reason. Detection of a cystic mass in relation to the appendix is diagnostic of mucocele in contrast-enhanced CT [11]. Some studies show that it is possible to differentiate malignant mucocele from a benign lesion by observing the radiological features of mucocele [19]. However, all the neoplasms may not have macroscopic features of a mucocele. A local study found that apart from mucocele, mucinous adenocarcinoma of the appendix can have macroscopic features like masses and irregular thickening of the wall [15]. In our case, contrast-enhanced computed tomography of the abdomen did not show an apparent change in the appendix. At the same time, intraoperatively, we did not observe a typical mucocele. It was a dilated tip, and the mucin was found within the lumen of the appendix. This means the tumour might have ruptured towards the mucosa of the appendix rather than confining it to the wall of the appendix. This rupture might have led to the bleeding into the lumen of the appendix. Therefore, the diagnosis of appendiceal mucinous adenocarcinoma may be made intraoperatively.

In our case, the suspicion was towards the appendix as the appendicular opening was filled with a blood clot mixed with mucinous material (Figure 1) in colonoscopy. This indicates that there is a possibility of an abnormal mucous plug over the appendicular opening during the colonoscopy in the patients with mucinous adenocarcinoma of the appendix.

Suppose an abnormal appendix is found intraoperatively, a thorough examination of the appendix by looking at the base of the appendix is needed. In that case, mesoappendix periappendicular tissues and peritoneal survey for the features of extravasated mucin or peritoneal deposits as a result of the ruptured appendix are warranted. Also, the presence of locoregional lymph node involvement should be looked for [11].

Surgical options of appendiceal mucinous lesions are controversial, being either to offer appendicectomy with excision of mesoappendix or right hemicolectomy (RHC). As Murphy et al. stated, if the size of the mucinous neoplasm is less than 2 cm and not involving the base of the appendix or mesoappendix, an appendicectomy with the removal of mesoappendix is accepted [11]. Resection should be adequate, and thorough washing of the peritoneal cavity has to be done. According to Murphy et al. and Choudry and Pai, for mucinous adenocarcinoma of >2 cm, it is recommended to do a right hemicolectomy as it may have lymph node metastasis [20]. Some studies show that there is a survival benefit when the right hemicolectomy is offered [21]. Nonetheless, a recent study by Turaga et al. found that the nodal metastasis is less in mucinous carcinoma of the appendix than nonmucinous carcinoma of the appendix and there is no survival benefit performing right hemicolectomy in known node-positive or metastatic disease [22]. In our case, right hemicolectomy was performed as the appendix was tightly adhered to the caecum, which is a macroscopic feature of invasion of adjacent organs. And the tumour size was approximately 2 cm.

All the caution should be taken to prevent any rupture of the lesion as it may lead to PMP later. It is recommended to convert a laparoscopic appendicectomy to an open procedure or make a large incision if it is difficult to retrieve the appendix [11, 20]. It is observed that PMP can occur even after ten years following resection of mucinous neoplasms of the appendix. If the mucinous appendiceal lesion is already ruptured but without features of extra appendiceal spread or ruptured during the surgery, it is recommended to do the right hemicolectomy. The removal of mucin pools and a meticulous peritoneal toilet is needed. Also, such patients should be followed up systematically following the surgery to detect recurrence with tumour markers like CEA, CA19-9, and CA125 and contrast-enhanced CT abdomen at least five yearly [11, 23]. For the mucinous appendiceal lesions which are ruptured and having features of PMP, it is recommended to manage the patient in a specialised centre for a definitive pathological review and decide on definitive management of complete cytoreductive surgery and hyperthermic intraperitoneal chemoperfusion (HIPeC) [20].

In summary, if the appendicular lesion has features of invasion radiologically and/or intraoperatively, right hemicolectomy is beneficial. For a benign-looking lesion, appendicectomy with excision of the mesoappendix is acceptable. All effort should be taken to prevent iatrogenic rupture of the mucinous appendix as it may lead to PMP later.

## 4. Conclusion

Mucinous adenocarcinoma of the appendix is a rare tumour, and presenting as a life-threatening severe gastrointestinal bleed is even rare, leading to diagnostic as well as therapeutic challenges. Initial patient stabilisation, thorough examination, investigation, and timely decision-making based on collective evidence lead to a better patient outcome.
